# Epigenetic profiling of human brain differential DNA methylation networks in schizophrenia

**DOI:** 10.1186/s12920-016-0229-y

**Published:** 2016-12-05

**Authors:** Sheng-An Lee, Kuo-Chuan Huang

**Affiliations:** 1 Department of Information Management, Kainan University, Taoyuan, Taiwan; 2 Department of Psychiatry, Beitou Branch, Tri-Service General Hospital, National Defense Medical Center, Taipei, Taiwan; 3 Department of Nursing, Ching Kuo Institute of Management and Health, Keelung, Taiwan

## Abstract

**Background:**

Epigenetics of schizophrenia provides important information on how the environmental factors affect the genetic architecture of the disease. DNA methylation plays a pivotal role in etiology for schizophrenia. Previous studies have focused mostly on the discovery of schizophrenia-associated SNPs or genetic variants. As postmortem brain samples became available, more and more recent studies surveyed transcriptomics of the diseases. In this study, we constructed protein-protein interaction (PPI) network using the disease associated SNP (or genetic variants), differentially expressed disease genes and differentially methylated disease genes (or promoters). By combining the different datasets and topological analyses of the PPI network, we established a more comprehensive understanding of the development and genetics of this devastating mental illness.

**Results:**

We analyzed the previously published DNA methylation profiles of prefrontal cortex from 335 healthy controls and 191 schizophrenic patients. These datasets revealed 2014 CpGs identified as GWAS risk loci with the differential methylation profile in schizophrenia, and 1689 schizophrenic differential methylated genes (SDMGs) identified with predominant hypomethylation. These SDMGs, combined with the PPIs of these genes, were constructed into the schizophrenic differential methylation network (SDMN). On the SDMN, there are 10 hypermethylated SDMGs, including GNA13, CAPNS1, GABPB2, GIT2, LEFTY1, NDUFA10, MIOS, MPHOSPH6, PRDM14 and RFWD2. The hypermethylation to differential expression network (HyDEN) were constructed to determine how the hypermethylated promoters regulate gene expression. The enrichment analyses of biochemical pathways in HyDEN, including TNF alpha, PDGFR-beta signaling, TGF beta Receptor, VEGFR1 and VEGFR2 signaling, regulation of telomerase, hepatocyte growth factor receptor signaling, ErbB1 downstream signaling and mTOR signaling pathway, suggested that the malfunctioning of these pathways contribute to the symptoms of schizophrenia.

**Conclusions:**

The epigenetic profiles of DNA differential methylation from schizophrenic brain samples were investigated to understand the regulatory roles of SDMGs. The SDMGs interplays with SCZCGs in a coordinated fashion in the disease mechanism of schizophrenia. The protein complexes and pathways involved in SDMN may be responsible for the etiology and potential treatment targets. The SDMG promoters are predominantly hypomethylated. Increasing methylation on these promoters is proposed as a novel therapeutic approach for schizophrenia.

**Electronic supplementary material:**

The online version of this article (doi:10.1186/s12920-016-0229-y) contains supplementary material, which is available to authorized users.

## Background

Schizophrenia is a complex mental illness, which is caused by the malfunctioning of many genes. There are multiple neurophysiological causes for its symptoms. Approximately 1008 schizophrenia-related genes have been identified by genetic studies such as GWAS and methods of systems biology, which provides a global view of genetics of the disease [1]. Besides, in the postmortem brain samples of schizophrenia patients, 4116 schizophrenia marker genes have been found to show different expression patterns [2]. Previous studies have mostly focused on the discovery of schizophrenia-associated SNPs, genetic variants and more recently, gene expression patterns. However, more and more studies started to investigate the differences in epigenetics of schizophrenia patients. Epigenetics can provide important information on how the environment effects the genetic structure of the disease [3]. By cross-referencing the disease-related SNPs, the differentially expressed genes and the epigenetic changes, we may reveal the insights of schizophrenia etiology.

Methylation of genomic DNA could mediate gene expression. Although there has not been any specific methylated gene patterns identified for schizophrenia, there are significant associations between promoter CG islands (CGIs) hypermethylation vs. the up-regulation of genes, and the hypomethylation vs. the down-regulation of genes [4]. CGIs have been suggested to suppress gene expression by blocking the promoters. There are significant differences between the overall and specific methylation levels of the different tissue samples of schizophrenia [5]. Recent analyses of the methylation arrays of postmortem brain samples indicated that the hypermethylation of RELN, hypermethylation and down-regulated transcription of SOX10, and hypomethylation of MB-COMT promoters may contribute to the development of schizophrenia [6]. Genes such as COMT and REELIN, as well as a few others in the dopaminergic, serotonergic and GABAergic pathways, have also shown differential methylation profiles in schizophrenia samples [7]. Differential methylation has been noted in specific schizophrenic candidate gene groups (e.g. RELN, BDNF, COMT, 5-HTT and glutamate receptor genes) [8]. Global hypomethylation has also been noted in schizophrenia patients in experiments with larger sample sizes [9].

There are also evidences of the environmental factors that altered the methylation of genomic DNA in schizophrenia. Lower LINE-1 methylation in peripheral blood leukocytes has been noted in first-episode schizophrenia patients with histories of childhood trauma [10]. Genome-wide DNA methylation profiling of peripheral leukocytes conducted with 24 drug-naïve schizophrenic patients and 23 healthy controls showed that methylated CpG sites were in 78.5% of the promotor regions in peripheral blood leukocytes [11].

The methylation and maintenance of CpG sites is crucial for neuron cell differentiation for the development of synaptic plasticity, learning ability and memory. Promotor hypermethylation of candidate genes in neurons is associated with transcriptional down-regulation of the corresponding mRNAs in postmortem studies of schizophrenia [12]. Differences in epigenetic patterns may contribute to phenotypic variations and disease susceptibilities [13]. Previous studies were mostly done on mouse models or stem cell lines [14, 15]. Nonetheless, a vast amount of methylation arrays of postmortem human brains have been released recently [16, 17]. These latest advances may implicate the importance of methylation patterns in schizophrenic patients. Most studies of gene methylation of mental diseases focused on the differential methylation patterns of genes. Little has been done on the correlations of differentially methylated genes and the expression of target gene.

Recent researches of schizophrenia tried to combine the methylation profiles of susceptible genes with network biology analyses. There are many studies attempted to locate differentially expressed schizophrenic genes on protein-protein interaction (PPI) networks and related pathways [2, 18–21]. Differentially expressed disease genes from postmortem brain samples of schizophrenia provide an overview of the disease maker genes. The analyses of disease PPI networks, the underlying pathways and protein complexes may construct a backbone for developing potential treatment strategy of the disease. Topologically and functionally important genes on the PPI networks may be seen as potential drug targets [19].

This study used a network biology approach to analyzed previous published DNA methylation profiles of the prefrontal cortex of 335 healthy controls and 191 schizophrenic patients [16]. In this dataset, there included 2104 CpGs identified as GWAS risk loci with differential methylation profile in schizophrenia [16]. Analyses of DNA methylation identified potential biological processes that regulate gene expression and contribute to disease mechanisms. We constructed the differential methylation and expression networks to interactions of methylated genes. Therefore, large scale analyses for differential methylation of schizophrenic susceptible genes were conducted and integrated with the differential expression data of schizophrenic susceptible genes to build the methylation–to-expression genetic network. The network explored the epigenetic mechanism of schizophrenic methylation networks, differential methylation pathways, complexes and corresponding biological functions. The genetic, epigenetic and transcriptomic information was integrated to give a comprehensive overview of schizophrenia.

## Methods

### Selection of significant genes with differential methylation

We downloaded published DNA methylation profiles of prefrontal cortex of 335 healthy controls and 191 schizophrenic patients from http://www.nature.com/neuro/journal/v19/n1/extref/nn.4181-S12.csv, with permission from Macmillan Publishers Ltd: Nature Neuroscience, Jaffe et al. [16], copyright 2016. The list compares differentially methylated CpGs of patients with schizophrenia to adult healthy controls. Methylated genes were identified in the brain samples from the NIMH Brain Tissue Collection at the National Institutes of Health, the Offices of the Chief Medical Examiners of Columbia and the Commonwealth of Virginia, Northern District. DNA methylation was assessed using the Illumina HumanMethylation450 (450 k) microarray, which measures CpG methylation across >485,000 probes covering 99% of Ref-Seq gene promoters.

Schizophrenia differential methylation analysis was made among 335 patients with schizophrenia diagnosed by DSM-IV and 191 controls. A Chi-squared test (df = 1) was performed to determine difference methylation significance. Hyper- or hypomethylation genes were calculated by moderated t-statistic values from 2104 CpGs. 1689 schizophrenic differential methylation genes (SDMGs) were selected. There were 688 differential methylated promotors, in which there were 16 hypermethylation promotors and 672 hypomethylation promotors.

The differentially expressed genes were collected from different schizophrenic candidate genes (SCZCGs) of differential expression gene databases from Huang, et al. [18], Huang, et al. [2] and Wu, et al. [22]. The significant differentially expressed transcripts with over- or under-expressed genes in the brain samples were selected by comparing the schizophrenia and control samples with Student t-tests. The corresponding *t*-test calculated the *p*-value for each gene. The genes with a *p*-value <0.05 were defined as SCZCGs. There are 615 over-expression genes and 1010 under-expression genes in SCZCGs. By exclusion of missing official symbols, a total of 1538 differential expression genes were selected for analysis.

For obtaining potentially involved pathways in schizophrenia, the pathway enrichment analysis of the significant differentially expressed genes are prioritized and the significance of corresponding pathways is ranked by *p*-value with FDR less than 0.05 using fdrtool [23].

### Differential genetic methylation network in schizophrenia

The schizophrenic differential methylation network (SDMN) was constructed for the comprehensive view of methylation profile in schizophrenia. The SDMN was generated by query-query protein-protein interaction (QQPPI) [24] and genetic interactions of SDMGs was recorded in version 8 of the Pathway Commons Database [25]. Pathway Commons Database [25] which collects BIND [26], BioGRID [27], CTD [28], DIP [29], HPRD [30], HumanCyc [31], IntAct [32], KEGG [33], NetPath [34], PANTHER [35], PhosphoSitePlus [36], PID [37], Reactome [38], SMPDB [39], TRANSFAC [40], MiRTarBase [41], DrugBank [42], Recon 2 [43], and WikiPathways databases [44] contain 34,661 molecular pathways.

The regulatory interactions and potential pathways of genes were investigated for understanding the disease mechanism of schizophrenia. To explore the modulation and regulatory relationships between the schizophrenic hypermethylated promotors and the differential expression genes, we identified the hypermethylated promotors and the Level 1 PPIs of SCZCGs to constructe the hypermethylation to differential expression network (HyDEN).

### Exploration of methylation profile and potential pathways

The biological features of schizophrenic methylation profile were discussed in this study. With the modularity of clique, complex and enrichment analysis of pathway, the bioinformatics analyses were performed for (a) the understanding of methylation profile interacted with differential gene expression in schizophrenia, (b) the discovery of potential cliques, complexes and involving pathways from enrichment analysis of SDMN, and (c) the evaluation of shared disease markers for schizophrenia and other major psychiatric disorders from PsyGeNET [45].

**Fig. 1:**
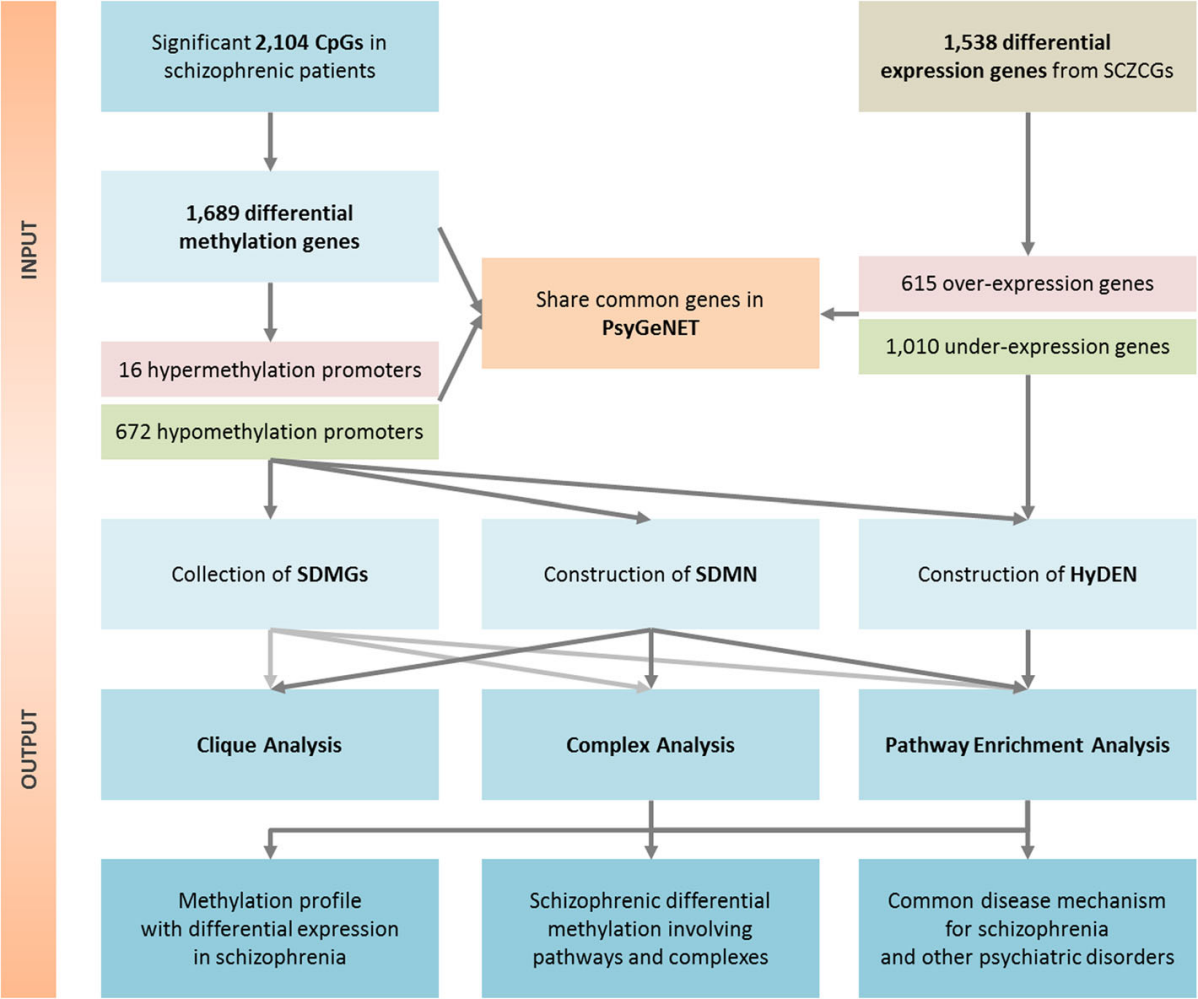
Flowchart for the analysis and exploration of disease methylation profiles and related pathways. The differentially methylated genes and differentially expressed genes were selected to construct the SDMN and HyDEN. The PsyGeNET also shares disease marker genes with the list of SDMGs and SCZCGs. By the analysis of cliques, functional complexes and enriched pathways, we (a) studied the methylation profiles with differential gene expression in schizophrenia, (b) discovered related pathways from enrichment analysis of schizophrenic differential methylation, and (c) identified shared disease mechanisms for schizophrenia and other major psychiatric disorders

The clique and complex analysis was performed with data of the CORUM database [46] which has a collection of experimentally verified mammalian protein complexes to reveal the corresponding cliques complexes from SDMN. The interaction networks were generated by Cytoscape [47]. The pathway enrichment analysis was performed by Integrated Pathway Resources, Analysis and Visualization System (iPAVS) [48]. It is an integrated biological pathway database designed to support pathway discovery in systems biology research of schizophrenia. The analytic flowchart of this study is shown as Fig. 1.

## Results

### Schizophrenic genes with differential methylation and expression

There are 1689 SDMGs with *p*-value <0.05 identified as listed in Additional file 1. The SDMGs and SCZCGs have 123 overlapped genes. The result illustrates the differential methylation and expression gene profile for schizophrenia (Additional file 1).

**Fig. 2:**
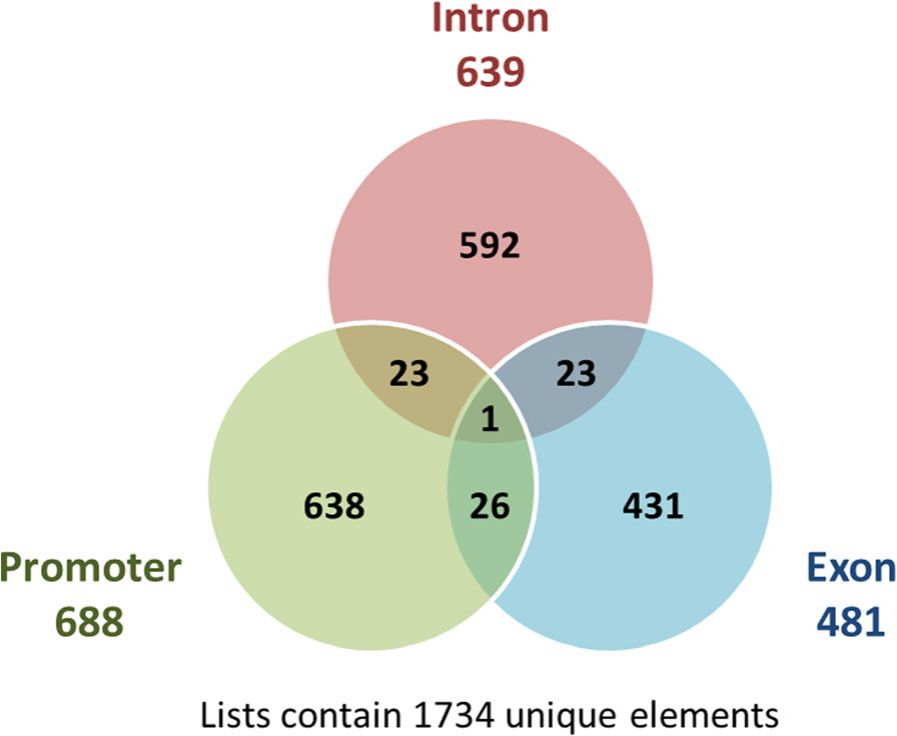
The Venn diagram for the distribution of methylation sites in promoters, introns or exons. This Venn diagram compared the numbers of methylation sites in promoters, introns and exons. There are 639 genes that are differentially methylated in intron, 688 genes in promoters and 481 genes in exons. Genes which have been more than one regions are 23 (intron and promoter), 23 (intron and exon) and 26 (promoter and exon), respectively. DAAM1 is differentially methylated is in promoter, intron and exon regions

There are 688 (39.6%) genes (16 hypermethylation/672 hypomethylation, ratio 2.38%) differentially methylated in promotor regions from 1869 schizophrenic differentially methylated genes. 639 (36.9%) genes (24 hypermethylation/615 hypomethylation, ratio 3.90%) are differentially methylated in introns. 481 (27.7%) genes (23 hypermethylation/458 hypomethylation, ratio 5.02%) are differentially methylated in exons. The Venn diagram revealed that the most differential methylation genes are in promotor regions (39.6%) and least differentially methylated in exons (27.7%) of the schizophrenic methylation profile on specific gene location. The very different methylation profile in promotor regions may an etiology of schizophrenia. Previous studies have shown evidences of DNA methylation profile of several common genetic loci in schizophrenia. In this study, the hypomethylation of promotors was involved in schizophrenia. There is one gene (DAAM1) which was hypermethylated throughout promotors, introns and exons. The Venn diagram is shown as Fig. 2.

### Topological analysis of SDMN

**Fig. 3:**
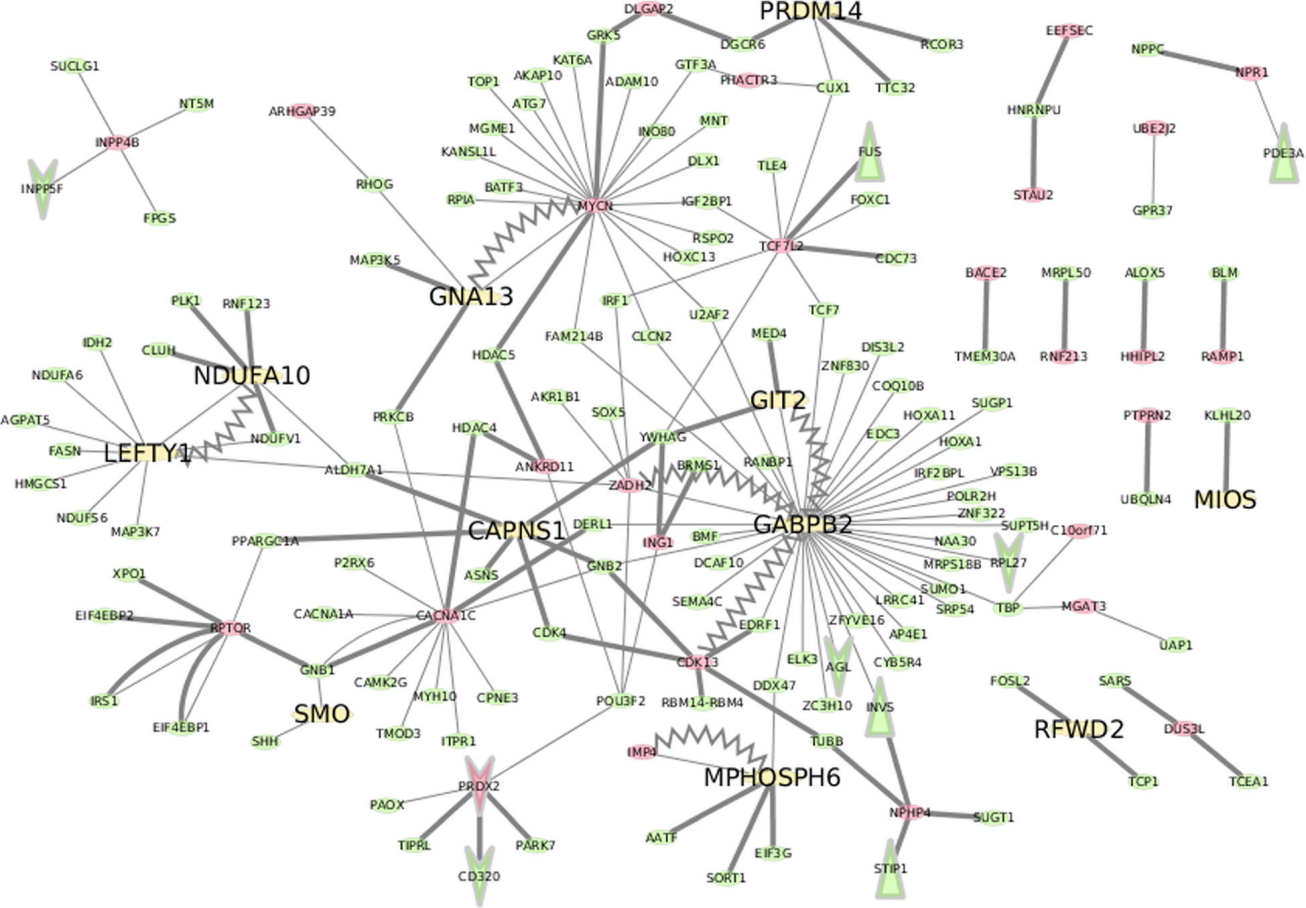
The differential methylation network of schizophrenia. The schizophrenic differential methylation network (SDMN) was generated by QQPPI of schizophrenic differential methylated genes (SDMGs) with underlying pathway enrichment. Among those SDMGs, there are 10 hypermethylated promoters in SDMN including GNA13, CAPNS1, GABPB2, GIT2, LEFTY1, NDUFA10, MIOS, MPHOSPH6, PRDM14 and RFWD2. They represent the key roles in modulating specific regulatory functions in schizophrenia. The thick lines represent the protein-protein interactions, the thin lines are binary relations (such as biochemical reactions) between genes in Pathway Commons. The zig-zag lines stand for both protein-protein interactions and binary relations. The green and red nodes stand for hypo- and hypermethylated genes respectively. The yellow nodes are depicted as hypermethylated promoters. The V-arrow nodes are depicted as under-expression genes and the triangular nodes are depicted as over-expression genes

The schizophrenic differential methylation network (SDMN) was generated by QQPPI and genetic interactions in Pathway Commons Database of SDMGs. Among SDMGs, there were 10 hypermethylated genes in SDMN including GNA13, CAPNS1, GABPB2, GIT2, LEFTY1, NDUFA10, MIOS, MPHOSPH6, PRDM14 and RFWD2. The hypermethylated genes are distributed throughout the SDMN and might be important in the regulatory inhibition of gene expression in schizophrenia. The inferring interaction network is shown in Fig. 3.

The 10 schizophrenic hypermethylated genes discovered by SDMN are associated with biological functions such as cell structure, energy metabolism, mitochondrial function, GABA metabolism, signaling transduction and zinc fingers. These schizophrenic hypermethylated genes may have vital roles in the etiology of schizophrenia. For example, GNA13, a candidate disease marker of schizophrenia according to GWAS studies [49], effects brain microstructure and maintenance of the white matter. It plays a crucial role for G-protein signaling and neurodevelopment [50]. Expression of CAPNS1 gene with signal transductions of calcium-dependent activity changes in schizophrenia [51, 52]. It also significantly contributes to platelet activity and thrombosis [53], which may contribute to the increased risk of thrombogenesis in schizophrenic patients [2]. From the interaction aspect of neurotransmission, calpain-mediated break down of fGAD65 results in decreased level of the GABA synthesis which leads to reduced GABA neurotransmission [54]. The mTOR has a role mediated by Wnt signaling pathway in the neuropathology of schizophrenia [55]. LEFTY1 gene encodes a member of the TGF-β family of proteins. Genetic variants in TGFB1 gene affect susceptibility to schizophrenia. TGF-β signaling might be a valid link contributing to schizophrenia patients [56, 57]. NDUFA10 has been contributed to the abnormalities of mitochondrial function in schizophrenia [58]. Also, the change of mitochondrial gene in respiratory electron transport chain responses to the exposure of antipsychotics [59].

Previous studies have validated the relationships between the hypermethylated genes and schizophrenia, yet, little is known about how methylation profile modulate the disease phenotype. With the analysis of SDMN, we could investigate the relationship between the hypermethylated genes and epigenetic mechanism in which the future experimental validation were needed. It may be one of the major disease mechanisms of schizophrenia.

The following hypermethylated genes are associated to schizophrenia susceptibility for the first time in this study, and have not been experimentally validated. PRDM14, a member of PRDM family and zinc finger proteins involving transcriptional regulators decrease [60]. Another gene, RFWD2 (COP1) is a cancer suppressor and effects human β-Cell Insulin Secretion [61]. Loss of COP1 expression may contribute to tumorigenesis and regulate the expression of tumor suppressor TP53 [18, 62, 63]. RFWD2 may indirectly influence the etiology for schizophrenia. MPHOSPH6 is related to the pathways in Sertoli-Sertoli Cell Junction Dynamics and Deadenylation-dependent mRNA decay; it is in a RNA exosome complex and is genomewide associated with SNP clusters in schizophrenia [64]. MPHOSPH6 also interacts with TP53, which has imporatnt biological functions in schizophrenia pathology [65]. GABPB2 has not yet established relationships with schizophrenia. However, GABPB2 may be involved in the nuclear control of mitochondrial function which is important to the pathogenesis for schizophrenia [2]. The above genes not only influence the genetic methylation levels, but also the gene expression.

### Hypermethylated genes interact with differential expression network (HyDEN)

The DNA hypermethylation could disable the gene function and decrease the gene expression level in many disease mechanisms, hence the relatioships between hypermethylated genes and differential expression was studied. All hypermethylated genes (or promoters) were collected and constructed into the HyDEN with the Level 1 PPIs of the encoded proteins.

**Fig. 4:**
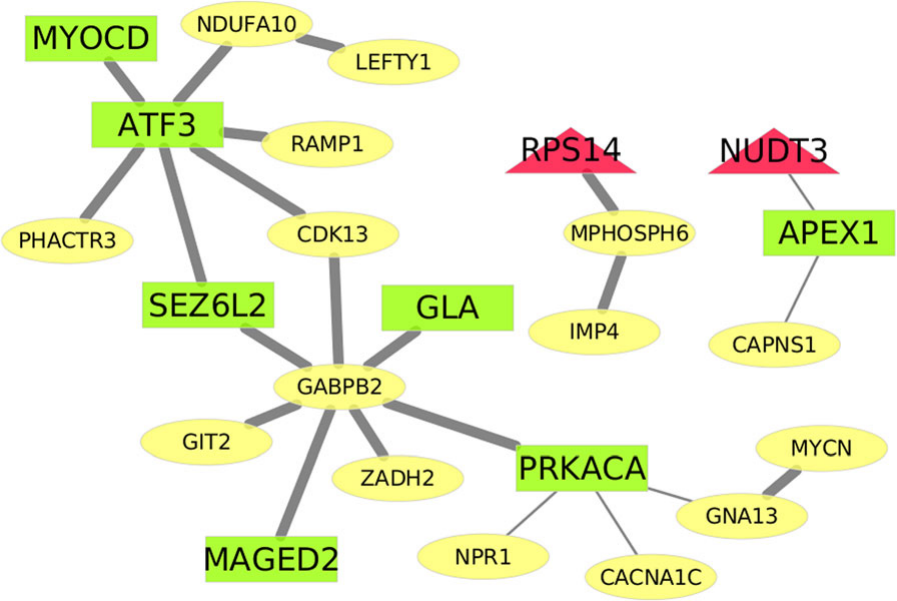
Hypermethylation to differential expression network. The hypermethylation to differential expression network (HyDEN) was constructed by hypermethylated promoters, SCZCGs and their Level one PPIs. The schizophrenic hypermethylated promoters, which interacting with differential expression genes, indicate the majority of hypermethylated genes suppressed the expression SCZCGs. The red triangular nodes are depicted as over-expressed genes and the green rectangle nodes are depicted as under-expressed genes, the yellow eclipse nodes are hypermethylated genes. The thick lines are PPIs. The thin lines are binary relations (e.g. biochemical reactions) between genes listed in Pathway Commons Databases

The interactions in HyDEN may implicate important regulation mechanisms in schizophrenia. In Fig. 4, the schizophrenic hypermethylated genes (yellow colored) linked to differential expression genes (red colored for over-expressed and green colored for under-expressed). The figure indicates the majority of hypermethylated genes are linked to under-expression of schizophrenic candidate genes. Only MPHOSPH6 interacts with over-expressed RPS14. The HyDEN is composed of the hypermethylated genes in promotor regions and the interacting SCZCGs. Hypermethylated genes including RAMP1, CDK13 and PHACTR3, suppressed the expression of ATF3. Hypermethylated gene GABPB2 suppressed the expression of SEZ6L2, GLA, PRKACA and MAGED2. Hypermethylated gene CAPNS1 suppressed APEX1. From the perspective of genetic interactions of DNA methylation and gene expression, HyDEN may have demonstrated the core disease mechanism of schizophrenia.

### Biological pathways with differentially methylated schizophrenic genes

Based on the topological analysis, the pathway enrichment analysis and underlying biological functions from differential methylation profile are proposed. There are 8510 pathways (out of the 34,661 pathways listed in Pathway Commons Database) which contain SDMGs.

There are 29 corresponding pathways with FDR-adjust *p*-value <0.05 found in enrichment analysis from SDMGs which may implicate the underlying disease mechanisms and characteristics for schizophrenia under the regulatory role of SDMGs. Top ranked pathways with FDR *p*-value <0.05 are TGF_beta_receptor, Pyrimidine metabolism, metabolic pathways, WNT pathway, folate biosynthesis, nicotinate and nicotinamide metabolism and purine metabolism. The pathway enrichment analysis may indicate the biological functions influenced by SDMGs. It could reveal the potential disease mechanism and novel therapeutic strategy for schizophrenia. These pathways are listed in Additional file 2_A. In order to explore the mechanism influenced by methylated promotors, the enriched pathways are listed in Additional file 2_B. For the investigation of how hypermethylating SDMGs regulate the corresponding pathways, the enriched pathways influenced by the hypermethylating SDMGs with Level 1 PPIs are listed in Additional file 2_C.

The top ranked pathways in Additional file 2_C, such as TNF alpha, PDGFR-beta signaling, TGF beta Receptor, VEGFR1 and VEGFR2 signaling, regulation of telomerase, hepatocyte growth factor receptor signaling, ErbB1 downstream signaling and mTOR signaling pathways, may be the key players in the symptoms of schizophrenia. Among these pathways, tumor necrosis factor alpha (TNF-α) is a cytokine product. Its primary role is the regulation of immune cells with biological functions of apoptotic cell death, and inhibition of tumorigenesis and viral replication. Dysregulation of TNF-α production may causes negative symptoms of psychosis and schizophrenia [66, 67]. Platelet-derived growth factor receptors (PDGF-R) are cell surface tyrosine kinase receptors. Its subunits -A and -B are important factors which regulate cell proliferation, cellular differentiation, cell growth and neuronal development. The genes for platelet-derived growth factor beta (PDGFB) and PDGFB receptor (PDGFBR) may be important in the pathology of schizophrenia through interacting with the DRD2/DRD4 and NMDA receptors [68]. It should be noticed that PDGFRB mRNA transcripts are significantly increased in postmortem brains of schizophrenic patients [69].

The Wnt pathway and the canonical pathway are targets of antipsychotic treatments. The most consistent abnormalities associated with antipsychotic response are Akt1, glycogen synthase kinase3beta and beta-catenin pathways [70]. VEGF is associated with inflammation reaction. Its serum levels are related to prefrontal cortex abnormalities in schizophrenia [71]. Shortened telomere length is found in unremitted schizophrenic patients [72]. A significant decrease is noted in telomerase activity among individuals with schizophrenia [73]. Hyperactivity of the epidermal growth factor receptor family (ErbB) is implicated in the pathophysiology of schizophrenia. ErbB receptor tyrosine kinases may be novel therapeutic targets for schizophrenia. Deficits in ErbB signaling pathway might contribute to the neurological development of psychiatric diseases [74, 75]. ErbB inhibitors appear to have anti-dopaminergic actions to alleviate behavioral symptoms in animal models for schizophrenia [76]. The mTOR signaling cascade involves in the regulation of neuronal morphology and synaptic plasticity. Disrupted mTOR signaling cause impaired function of protein synthesis in schizophrenia [55]. Those involved pathways are significantly found by enrichment analysis of differential methylated genes with hypermethylated genes and promotor regions. They could reveal the pathology and disease mechanism for schizophrenia.

### Shared disease mechanisms of schizophrenia and other psychiatric disorders

In order to compare the common disease mechanism between schizophrenia and the different psychiatric disorder, a knowledge platform for the exploratory analysis of psychiatric diseases and their associated genes known as PsyGeNET [47] was analyzed to search for differential methylated genes of schizophrenia. The platform focuses mainly on major depression, and alcohol and cocaine additions.

There are 308 overlapped genes in PsyGeNET from 1538 SCZCGs, and 64 overlapped genes from 688 SDMGs in promotors and PsyGeNET. The selected genes are listed in Additional file 3. Of the 308 overlapped genes from PsyGeNET and SCZCGs, the most frequently appeared (> = 7 psychiatric disorders) genes are DRD2, TPH2, S100B, GAD1, DTNBP1, GFAP and CARTPT among different psychiatric disorders such depressive disorder, bipolar disorder, alcoholism, cocaine-related disorders and suicide. It reveals that those vital mental illnesses in PsyGeNET may share common marker genes with the SCZCGs. The molecular mechanisms for those genes involves G-protein receptor activity, tryptophan metabolism, cell cycle progression and differentiation, GPCR signaling, glutamate metabolism, neurotransmitter release, actin cytoskeleton reorganization*,* structural molecule activity and energy balance. They are also pivotal genes in the development of schizophrenia, bipolar disorder, major depressive disorder, cocaine dependence and Alzheimer’s disease [77–84].

Of all 64 overlapped genes from the SDMGs and PsyGeNET, the most frequent appeared genes are NR3C2, HDAC5, FTO, XBP1, RNF41 and NDUFV1. Those are pivotal genes for alcoholism, schizophrenia, major depression, bipolar disorder [85–90]. The involved biological functions of transcription factor activity, steroid hormone receptor activity, transcriptional regulation, cell cycle progression and developmental events, oxidative RNA demethylase activity and NADH dehydrogenase (ubiquinone) activity. The shared pathways are signaling by GPCR, immune system and metabolism.

### Protein complexes and genetic interactions in SDMGs and SCZCGs

In order to understand the involved protein complexes in schizophrenia of how SDMGs interact with the expression level of SCZCGs, we searched CORUM for the potential protein complexes responsible for the regulation and epigenetic mechanism in schizophrenia. CORUM is a database that provides information of experimentally characterized protein complexes from human resource to predict the occurrence of protein complexes in different polygenetic groups [91]. We analyzed the gene groups from SDMGs and SCZCGs, and searched against CORUM to find the potential protein complexes related to the etiology of schizophrenia from alteration of DNA methylation. The protein complexes involved in SDMGs and SCZCGs are listed in Additional file 4.

The most important protein complexes involved in SDMGs and SCZCGs may include Nop56p-associated pre-rRNA complex, ribosome related subunit, mitochondrial respiratory chain complex I, TFTC complex and PCAF complex. The biological functions of those complexes are associated with ribosome biosynthesis, mitochondrial dysfunction and pre-rRNA processing. However, the top ranked complexes represented in SDMGs including SMCC complex, Mediator complex, Nop56p-associated pre-rRNA complex, CDC5L complex, CF IIAm complex and 55S mitochondrial ribosome complex. These complexes are translated by aberrant SDMGs to perform specific protein functions, which might be the potential molecular mechanism in epigenetic regulation for schizophrenia. The inheritable alterations of these complexes might explain the roles of hereditary factors in the etiology of schizophrenia with DNA methylation [92].

## Discussion

### The roles of DNA differential methylation in schizophrenia

External factors such as environmental stress are known to cause the onset of schizophrenia. Exposure to stress induces stable changes by transcriptional dysfunction, resulting in aberrant changes of genetic expression, neural circuit functions and ultimately, behavior changes and disease symptoms [93]. Epigenetic factors such as aberrant DNA methylation have important roles in regulating gene expression [94]. Epigenetic changes may be one of the pivotal features of many human mental disorders. In cancer etiology, the promoter hypermethylation also plays a major role by aberrant transcription of critical regulator genes such as tumor suppressor genes with the implications for the hypomethylation factors in the novel treatment strategy of cancer [95].

Epigenetic mechanism produces DNA methylation which alter gene expression without altering underlying DNA sequence. Epigenetic changes may be passed on for multiple generations by cell division [96]. Evidences of linkage analysis in schizophrenic family suggest a hereditary susceptibility [97]. The methylation of DNA confers long-term epigenetic silencing which could be reprogrammed by demethylation of DNA repair [98]. It is implicated that the epigenetic change, especially from the differentially expression genes, regulate the methylation of SDMGs and the production of corresponding protein complexes.

Methylation in the transcribed region is often correlated with expression, and high levels of gene expression is often associated with low promoter methylation [99–101]. The hypermethylated genes are usually under-expressed. The relationships between the hypermethylated genes and the differential expression genes in schizophrenia (HyDEN) reveals that the hypermethylated promotors are not necessarily associated with their differential expression level, instead, the interacting genes with hypermethylated SDMGs are mostly under-expressed SCZCGs. These gene interactions may be important in the regulation of corresponding pathways and biological functions for schizophrenia.

Our results suggest that the hypomethylated genes are predominant in schizophrenia. Reducing hypomethylation of SDMGs or SCZCGs could be a novel therapeutic treatment method for schizophrenia. There might be the protective factors as per the etiology of cancer [18], in which most promotors are hypermethylated. Some hypermethylating agent, such as vitamin B1, could induce up-regulation of methyltransferase and reversion of hypomethylation as an adjuvant treatment in schizophrenia [102]. It has postulated that deficiency of vitamin B1 may result in genetic methylation and biochemical lesion relating to neurotransmitter metabolism in brain, leading to psychotic manifestations [103].

**Fig. 5:**
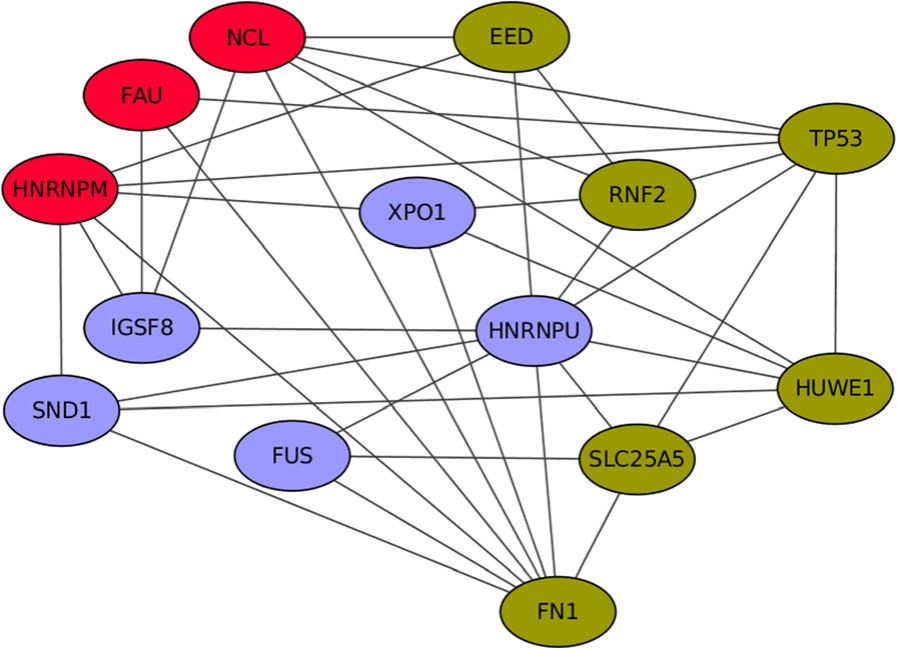
The ribosome-associated PPI network. The ribosome-associated PPI network constructed with SDMGs and SCZCGs revealed the regulatory role of epigenetic mechanism in schizophrenia. The red and green nodes are depicted as over- and under-expressed SCZCGs, respectively. The purple nodes are hypomethylated promoters. The ribosome biosynthesis is likely to be regulated by hypomethylated SDMGs which interacts with differentially expressed SCZCGs. The regulation of ribosome expression by the hypomethylated promoters may be crucial in the pathogenesis for schizophrenia

DNA methylation and histone modifications can alter genome functions under exogenous influences. These heritable changes in gene expression may be more than just changes of DNA sequence. The ribosome-associated PPI network was constructed with SDMGs combined with SCZCGs (Fig. 5), and the topological analysis revealed that groups of hypomethylated genes XPO1, HNRNPU, IGSF8, SND1 and FUS which ingeniously interact with over-expressed genes NCL, FAU and HNRPNPM, as well as with under-expressed genes: EED, TP53, RNF2, HUWE1, SLC25A5 and FN1. It reveals the regulatory role and epigenetic mechanism in schizophrenia with novel targets of therapeutic agents. In the analysis of HyDEN and the regulatory hypomethylated genes in ribosome, there appears to have interactions between differential DNA methylation and differential expression levels. There seems to be interactions among the regulatory factors at different stages of gene expression and genetic inheritance.

In published works, the hypermethylated genes in HIPPO signaling and MAPK signaling pathways have been observed in schizophrenic patients. Hypomethylated promoters in CREB signaling, dopamine-DARPP32 feedback in cAMP signaling and Ephrin receptors have also been noted. CREB3L2 in differential expression genes was under-expressed; CREB3 and PPP1R1B (DARPP32) were hypomethylated in SDMGs. Another example of hypomethylation in FAM63B was found in bipolar disorder. FAM63B was an epigenetic risk gene for schizophrenia and bipolar disorder [104]. However, FAM65B, FAM64A and FAM69A have been reported as hypomethylated in SDMGs. LRRTM1 hypomethylation in the promoter was reported as a risk factor for the development of schizophrenia [105]. LRRTM2 was over-expressed in Wu et al. [20]; LRRTM4 was under-expressed in Huang, et al. [2]. Antipsychotic such as quetiapine decreased the DNA methylation level of the promoter region of SLC6A4. Mood stabilizers could also reverse the hypermethylation process of CpG sites of SLC6A4 to be hypomethylated in bipolar disorder [106, 107]. However, SLC6A9 was under-expressed gene in Huang et al. [2].

### Epigenetics in protein complexes for schizophrenia

For the understanding of epigenetic mechanism for schizophrenia, the potential protein complex interactions reveal the relationships between the genomic and the environmental factors during disease development. Among the top ranked complexes, there are reported complexes by literature associated with epigenetic mechanism for schizophrenia. The mediator complex is a key role in regulation of transcription of RNA polymerase II. It was characterized as TRAP complex that facilitates transcriptional activation by thyroid hormone receptor [108, 109]. The mutations in TRAP230 could result in the attenuated functions by p53 activation to target RNA polymerase II [110] and transcriptional activators by TRAP/Mediator complex correlate with the development of schizophrenia [111, 112]. CDC5L complex is essential for the catalytic step of pre-mRNA splicing [113]. Epigenetic factors such as stress induced prefrontal cognitive dysfunction, schizophrenic patients with DISC1 mutations are vulnerable to the effects of stress by increasing cAMP levels [114]. DISC1 network of PPIs involved CDC5L indicates the protein or complexes that have been linked to schizophrenia [115]. It implicates that the DISC-1 associated CDC5L complex may explain the epigenetic mechanism in stress-induced prefrontal dysfunction in schizophrenia.

The alteration of mitochondria and dysregulation of energy metabolism in postmortem brain samples may contribute to implication of schizophrenia [116, 117]. The genetic interactions among mitochondrial genes and many under-expressed SCZCGs indicate the genetic predisposition of mitochondria dysfunction in schizophrenia. The genetic interactions between mitochondria and schizophrenia may be revealed by the DRD2-NDUFS7 and the FLNA-ARRB2 interactions [2]. In this study, NDUFA10 in HyDEN has found to be associated with the abnormalities of mitochondrial function in schizophrenia [58]. It plays a key role in respiratory electron transport chain responded to the exposure of antipsychotics [59]. NDUFA10 mutation causes mitochondrial complex I deficiency. It is associated with the progressive neurodegenerative disease such as Leigh syndrome [118], which possibly shares the same etiopathogeny with schizophrenia [119]. The mechanism involving NDUFA10 could be novel targets for schizophrenic therapeutic treatments.

## Conclusions

In order to understand the regulatory role of SDMGs, the epigenetic profiles of DNA differential methylation in schizophrenic postmortem brain samples was analyzed in this study. Aberrant DNA methylation have been associated with various neurodevelopmental and neuropsychiatric disorders. The interactions between SDMGs and SCZCGs may be part of the underlying disease mechanism of schizophrenia, with the DNA differential methylation influencing the levels of gene expression. It is implicated that corresponding complexes and pathways are pivotal and for the disease mechanism for schizophrenia.

The SDMN was extended with the Level 1 PPIs of SDMGs to explore the potential protein complexes and biochemical pathways of the disease mechanism and therapeutic options for schizophrenia. The SDMGs interacted with SCZCGs in a coordinated fashion in schizophrenia. The majority of hypermethylated SDMGs were associated with the under-expression of SCZCGs, indicating the regulatory role of hypermethylated promotors to suppress SCZCGs and the consequent suppressiong of coppresponding pathways such as TNF alpha, PDGFR-beta signaling, TGF beta Receptor, VEGFR1 and VEGFR2 signaling, regulation of telomerase, hepatocyte growth factor receptor signaling, ErbB1 downstream signaling and mTOR signaling. The dysfunction of these pathways may be the causes of schizophrenia. Altered DNA methylation, including both hyper and hypo, appears to play a complementary role in schizophrenia development. The predominant hypomethylation of SDMG promoters is distinct from the characteristic predominantly hypermethylation of promotors in cancers. Increasing methylation of these abnormally hypomethylated promotors could be a potential therapeutic option for schizophrenia.

## Additional files

Additional file 1: Differentially methylated genes with differential expression databases. (XLSX 170 kb)
Additional file 2: Pathway enrichment analysis of SDMGs, hypermethylating SDMGs and SDMGs with level 1 PPI. (XLSX 576 kb)
Additional file 3: The shared common genes of 688 SDMG promoters and PsyGeNET. (XLSX 20 kb)
Additional file 4: The protein complexes involved in SDMGs and SCZCGs. (XLSX 27 kb)
